# Treatment and disease progression in a birth cohort of vertically HIV-1 infected children in Ukraine

**DOI:** 10.1186/1471-2431-10-85

**Published:** 2010-11-23

**Authors:** Saboura Mahdavi, Ruslan Malyuta, Igor Semenenko, Tatyana Pilipenko, Claire Thorne

**Affiliations:** 1 MRC Centre of Epidemiology for Child Health, UCL Institute of Child Health, University College London, London, UK; 2 Perinatal Prevention of AIDS Initiative, Odessa, Ukraine

## Abstract

**Background:**

Ukraine has the highest HIV prevalence (1.6%) and is facing the fastest growing epidemic in Europe. Our objective was to describe the clinical, immunological and virological characteristics, treatment and response in vertically HIV-infected children living in Ukraine and followed from birth.

**Methods:**

The European Collaborative Study (ECS) is an ongoing cohort study, in which HIV-1 infected pregnant women are enrolled and followed in pregnancy, and their children prospectively followed from birth. ECS enrolment in Ukraine started in 2000 initially with three sites, increasing to seven sites by 2009.

**Results:**

A total of 245 infected children were included in the cohort by April 2009, with a median age of 23 months at most recent follow-up; 33% (n = 77) had injecting drug using mothers and 85% (n = 209) were infected despite some use of antiretroviral prophylaxis for prevention of mother-to-child transmission. Fifty-five (22%) children had developed AIDS, at a median age of 10 months (IQR = 6-19). The most prevalent AIDS indicator disease was *Pneumocystis jiroveci *pneumonia (PCP). Twenty-seven (11%) children had died (median age, 6.2 months). Overall, 108 (44%) children had started highly active antiretroviral treatment (HAART), at a median 18 months of age; median HAART duration was 6.6 months to date. No child discontinued HAART and 92% (100/108) remained on their first-line HAART regimen to date. Among children with moderate/severe immunosuppression, 36% had not yet started HAART. Among children on HAART, 71% (69/97) had no evidence of immunosuppression at their most recent visit; the median reduction in HIV RNA was 4.69 log_10 _copies/mL over a median of 10 months treatment. From survival analysis, an estimated 94%, 84% and 81% of children will be alive and AIDS-free at 6, 12 and 18 months of age, respectively. However, survival increased significantly over time: estimated survival rates to 12 months of age were 87% for children born in 2000/03 versus 96% for those born in 2004/08.

**Conclusion:**

One in five children had AIDS and one in ten had died. The half of children who received HAART has responded well and survival has significantly improved over time. Earlier diagnosis and prompt initiation of HAART remain key challenges.

## Background

Prevalence of HIV infection in Ukraine is 1.6% overall, with antenatal prevalence of 0.52%, the highest in Europe and the country is facing one of the fastest growing epidemics in the world[1]. According to UNAIDS estimates, 1.5 million people will be living with HIV/AIDS in Ukraine by 2010, two-thirds of whom will be of reproductive age [2]. The number of HIV infected pregnant women delivering in Ukraine has been increasing annually, reaching 5000 in 2007[3]. In Ukraine, the national Prevention of Mother to Child Transmission (PMTCT) Programme was implemented in 2001[2]. This programme has been successful with MTCT rates declining from above 25% in 2001 to 7% in 2006[1,4]. In 2007 the government implemented more comprehensive HIV/AIDS programmes and policies to scale up access to prevention, diagnosis and treatment services nationally, but antiretroviral therapy (ART) coverage for adult population remains low, with 35% of individuals with advanced HIV infection receiving ART in 2007 [5,6].

In children, recent studies have demonstrated that early diagnosis of HIV infection followed by early initiation of ART significantly reduces HIV related morbidity and mortality and has resulted in substantially improved survival [7-10]. This, together with the higher risk of clinical progression for vertically-infected infants compared with older children and adults, due to high HIV-1 RNA viral load (VL) and immature immune systems[7-9,11-16], reflects the essential need for scaling up access to paediatric HIV care and treatment.

Few data are available on the natural and treated history of vertically infected children in Eastern Europe. This information, including treatment patterns, clinical, immunological and virological impact of treatment and long-term outcomes of HIV infection in children, is important to achieve a better understanding of disease progression in this setting and for health care planning and decision-making related to therapeutic strategies. Our objective was to describe the clinical, immunological and virological characteristics, treatment and response in vertically-infected children living in Ukraine and followed from birth in an ongoing cohort study.

## Methods

The European Collaborative Study (ECS) is an ongoing cohort study, in which HIV-1 infected pregnant women are enrolled and followed in pregnancy, and their children prospectively followed from birth. The ECS was established in 1985 in Western Europe and centres from Ukraine first joined in 2000. ECS enrolment in Ukraine started with three sites (Odesa, Mykolaiv and Simferopol), increasing to six sites in 2006 (addition of Kyiv, Donetsk and Mariupol) and with the addition of Kryvyj Rih in 2009. All pregnant women are screened for HIV-1 infection at first antenatal visit during their pregnancy (routine, universal opt-out system) with repeat testing in the third trimester for women testing negative. A policy for rapid HIV testing during labour for women with undocumented HIV status started in 2003 for those not having antenatal care. Pregnant women diagnosed with HIV infection before pregnancy, during pregnancy or intrapartum are offered to participate in ECS study, with informed consent. Standard questionnaires which are coded anonymised are used for collection of pregnancy, delivery and follow-up data. Infants are followed up at HIV/AIDS Centres until a diagnosis is made. HIV-1 infected children are then followed up ideally at least twice a year. Infected infants were diagnosed based on persistence of HIV antibody beyond age 18 months up until the start of 2006; subsequently, early diagnosis of HIV-exposed infants with DNA PCR testing was introduced nationwide, with facilities in three inter-regional laboratories. Data collected included maternal clinical and sociodemographic status, PMTCT intervention, delivery data, infant demographic and clinical characteristics, ART, other prophylaxis and laboratory markers of disease progression. Flow cytometry became available in Odessa and Simferopol in 2004; Donetsk and Kiev had this capacity from their first enrolments in autumn 2006.

Definition of HIV-1 infection was based on the development of AIDS and HIV-associated morbidity and mortality, persistence of HIV antibody beyond 18 months of age or detectable virus in two or more blood samples. CD4 counts were expressed as absolute counts, which vary with age. Children were therefore assigned to a Centres of Disease Control and Prevention (CDC) immunological category on the basis of their age and CD4 count at the time of each measurement. CDC immunological categories are as follows: normal (CDC immunological category 1), moderate immune suppression (category 2) and severe immune suppression (category 3) [17,18]. HIV RNA levels were measured with a lower limit of quantification of 400 copies/ml and thus undetectable VL was defined as VL less than 400 copies/ml[10,19].

### Statistical analysis

Kaplan-Meier survival analyses were carried out to estimate the proportion of children progressing to death and AIDS over time since birth (i.e. time of infection), separately and as a compound outcome variable. In addition, children born in 2000-2003 and in 2004-2008 were compared in terms of time to AIDS or death using Kaplan-Meier plots and the log rank test.

Data were entered and managed in an Access database using Microsoft Access XP (Redmond, WA, USA). Data analyses were carried out using STATA, (STATA Version 10; STATA Corporation, College Station, Texas, USA).

#### Ethics approval

The ECS has been approved by the Great Ormond Street Hospital for Children NHS Trust/Institute of Child Health Ethics Committee.

## Results

### Maternal and infant characteristics and PMTCT interventions

A total of 245 HIV-1 infected children were included in the cohort by April 2009 with a median age of 23 months (IQR, 14-46 months) at most recent follow-up visit. Most mothers were Ukrainian nationals (240/245, 98%) and either married (97/241, 40%) or cohabiting (101/241, 43%). Median maternal age at delivery was 27 years (IQR, 23-30) and for half of the mothers (n = 133) this delivery was their first live birth. A large proportion of mothers reported no specific risk factors for HIV acquisition but of the 132 who did, 80% (n = 106) noted sexual risk factors, of whom half also had an injecting drug use (IDU) history (Table 1). Overall, 15 (6%) infants had neonatal abstinence syndrome. The mothers of nine (4%) infected children were known to have died.

**Table 1:** Maternal and delivery characteristics

	N (%)
**Maternal risk factors for acquisition of HIV (n = 241)**	
IDU	24 (10)
Sexual	53 (22)
IDU & sexual	53 (22)
Other	2(0.8)
Not specified	107 (44)
**Time of maternal HIV diagnosis (n = 239)**	
Before pregnancy	62 (26)
During pregnancy	134 (56)
At delivery	43 (18)
**Mothers' WHO clinical stage at enrolment (n = 213)**	
Stage 1	92 (43)
Stage 2	110 (52)
Stage 3	8 (4)
Stage 4	3 (1)
**Mode of delivery (n = 245)**	
Vaginal	183 (75)
Emergency caesarean section	9 (3.5)
Elective caesarean section	53 (21.5)
**Gestational age (n = 245)**	
< 34 weeks	17 (7)
34-36	26 (11)
≥ 37	202 (82)
**Birth weight (n = 245)**	
< 2500 g	58 (24)
≥ 2500 g	187 (76)

A quarter (n = 63, 26%) of mothers had received no antenatal antiretroviral (ARV) prophylaxis for PMTCT, 27% (n = 67) had received zidovudine (ZDV) monotherapy, 20% (n = 50) intrapartum single dose nevirapine (sdNVP) only, a quarter (n = 61) ZDV with sdNVP and 2% (n = 4) had received antenatal HAART. Most neonates received ARV prophylaxis (Table 2) and thus the majority of children (n = 209, 85%) were infected despite the use of some PMTCT prophylaxis. Overall, around a fifth of children were delivered preterm (i.e. < 37 weeks gestation) (Table 1) and median birth weight was 2900 g (range, 1150-5000). The vast majority of children were bottle fed (237/244, 97%) with breastfeeding reported for seven children, three of whom were breastfed for a month and two were breastfed for 12 and 24 weeks, respectively.

**Table 2:** Children's characteristics

	N (%)
**Sex (n = 241)**	
Female	121 (50)
Male	120 (50)
**Care setting at most recent follow-up (n = 238)**	
Parental	196 (82)
Institutional	41 (17)
Adopted/Fostered	1 (0.4)
**Neonatal prophylaxis regime (n = 245)**	
Only sdNVP	76 (31)
Only ZDV	52 (21)
ZDV + sdNVP	77 (31)
ZDV + 3TC ± sdNVP	4 (2)
None	36 (15)
**Children's WHO clinical stage at most recent follow-up (n = 245)**	
Stage 1	120 (49)
Stage 2	49 (20))
Stage 3	27 (11)
Stage 4	49 (20)
**Deaths**	27 (11)

Coverage of infants with early virological diagnostic testing in this cohort was low, reflecting the fact that DNA PCR testing only became available in 2006: overall, 132 (54%) children had one or more DNA PCR tests and of these, 18 (14%) received their first virological test before age 4 months, 83 (63%) between 4-18 months and 31 (23%) after 18 months. Among children born in 2007/08 with PCR tests carried out, 23% (n = 8/35) tested before age of 4 months. Median age of children at first PCR test was 12.5 months (range, 0-88 months). Overall, median age of children at first DNA PCR test was 12.5 months (range, 0-88 months). The remaining 113 children without DNA PCR test results were diagnosed on the basis of antibody testing (100, 41%) after age 15-18 months or the development of AIDS (13, 5%).

### Treatment

**Figure 1:**
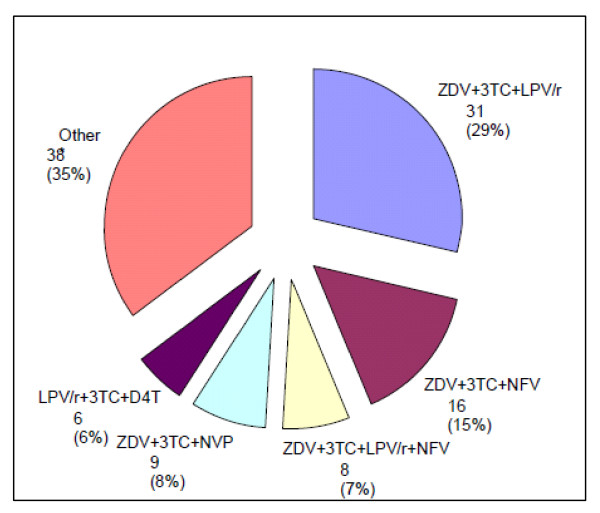
**Type of first line HAART regimen among 108 treated children**. Footnote: Other regimens: 3TC+ABC+LPV/r, ABC+ZDV+ddI, ddI+ZDV+LPV/r, ZDV+3TC+EFV, D4T+ddI+NFV, D4T+3TC+NFV, 3TC+ddI+LPV/r

Nearly half of the children (n = 108, 44%) had started highly active antiretroviral treatment (HAART) by their most recent follow-up visit. Most had initiated HAART within 2007 to 2009 (n = 91, 86%). The first-line HAART regimens are shown in Figure 1 with ZDV+3TC+LPV/r being the most common. Median age at HAART initiation was 18 months (IQR, 9-45 months) overall, but this decreased substantially over time, from 55 months for those born before 2004 to 9 months for those born in 2007-2008. Of children born in 2006/09, 88% (39/44) started HAART at < 18 months of age, substantially more than those born in 2000/2 and in 2003/4 (17% [4/20] and 20% [10/50] respectively) (*p *< 0.001). Median HAART duration at most recent follow-up was 6.6 months. No child had discontinued HAART and the majority (n = 100, 92%) remained on their first-line HAART regimen to date.

A total of 75 (31%) children had received prophylaxis at any time during follow-up, mainly trimethoprim (n = 71, 95%), with 9 (4%) children had received isoniazid as TB treatment/prophylaxis.

### HIV disease progression

Overall, 15.5% (38/245) of infants progressed to AIDS or death in their first year of life. A total of 55 (22%) children had developed AIDS by most recent follow-up, two of whom had not received HAART. Median age of progression to AIDS was 10 months (IQR = 6-19). The most prevalent AIDS indicator diseases were *Pneumocystis jiroveci *pneumonia (PCP), recurrent severe bacterial infection and HIV encephalopathy (Table 3). CD4 counts were available for 155 (63%) children, with a total of 351 measurements by last follow-up. Median age at first CD4 count measurement was 14.7 months. HIV RNA measurements were available for 132 (54%) children with 238 measurements in total to date. Table 4 shows CDC immunological categories at most recent follow-up stratified by age. Overall, three quarters of children (111/155) showed no evidence of immune suppression at this time, 17% (n = 27) had evidence of moderate immune suppression and 11% (n = 17) were severely immunosuppressed. Of the 44 children either moderately or severely immuno-suppressed, 16 (36%) had not received ART by their most recent follow-up visit. A total of 27 (11%) children had died, at a median age of 6.2 months (IQR 4-19); the most common causes of death were septicaemia (n = 13) and pneumonia (n = 8). Most (19, 75%) had progressed to AIDS before their death, of whom 14 (74%) died in their first year of life; 7 children died without receiving HAART. The overall mortality rate was 4.29 deaths per 100 child-years of follow-up.

**Table 3:** Prevalence of AIDS indicator diseases among 55 children with AIDS

	N (%)
Pneumocystis jiroveci pneumonia (PCP)	15 (27)
2+ severe bacterial infection within a 2-year period	13 (24)
HIV encephalopathy	9 (16)
Extra pulmonary m. tuberculosis infection	7 (13)
Opportunistic infection (unspecified)	6 (11)
HIV wasting syndrome	2 (4)
Weight loss > 10% of baseline	2 (3.5)
Candidiasis (oesophageal, bronchial or pulmonary)	1 (2)

**Table 4:** Immunosuppression categories at most recent follow-up visit (N = 155)

Immunologic category	Age of child at most recent follow-up visit					
	< 12 months		1-5 years		6-12 years	
	n = 35		n = 94		n = 26	
	μL	N (%)	μL	N (%)	μL	N (%)
1: No evidence of suppression	> 1500	20 (57)	> 1000	69 (73)	> 500	22 (85)
2: Evidence of moderate suppression	750-1499	7 (20)	500-999	19 (20)	200-499	1 (4)
3: Severe suppression	< 750	8 (23)	< 500	6 (7)	< 200	3 (11)

### Response to treatment

Half of the children on HAART (53/108) had been initiated on this recently and did not yet have a reported VL after HAART initiation. Of the 55 children with VL measurements available after HAART initiation, 71% (n = 39) had achieved an undetectable VL to date. In 46 children on HAART with both pre and post HAART VLs available, the median reduction in HIV-1 RNA was 4.69 (IQR 2.11-5.89) log_10 _copies/mL over a median duration of 10 months treatment (IQR, 5-38). Of children with a CD4 measurement available at HAART initiation or within 12 weeks prior to initiation, 41% (23/56) were either moderately (CDC immunological category 2) (n = 15) or severely (CDC immunological category 3) (n = 8) immunosupressed. Of the 97 (90%) children on HAART with a CD4 measurement at their most recent follow-up, 69 (71%) had no evidence of immunosuppression.

**Figure 2:**
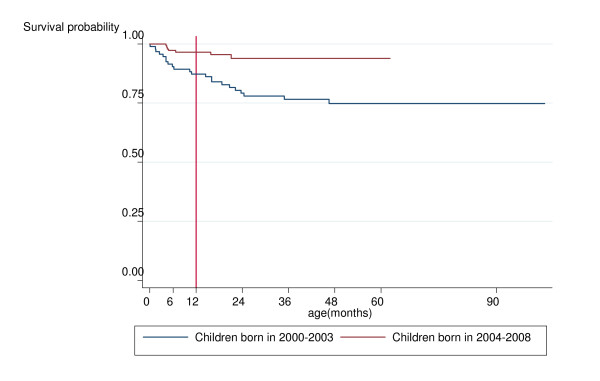
**Estimated progression to death, by time period of delivery among 94 HIV-1 infected children born in 2000 to 2003 and 151 born in 2004 to 2008**.

From Kaplan-Meier survival analyses, an estimated 94%, 84% and 81% of children will be alive and AIDS-free at 6, 12 and 18 months of age, respectively. A further analysis of time to progression to AIDS alone indicated that an estimated 4%, 13% and 17% of infected children will have developed AIDS by 6, 12 and 18 months of age. A further survival analysis stratified by calendar period indicated that progression to death decreased significantly over time (*p *< 0.001) (Figure 2): an estimated 87% and 84% of children born in 2000/03 survived to ages 12 and 18 months respectively, compared with 96% and 95% for children born in 2004/08. However, there was little change in AIDS-free survival rates over time, with estimated AIDS-free survival rates in children born in 2004/08 of 85% and 80% at 12 and 18 months versus 83% and 79% for those born in 2000/03. Estimated AIDS-free survival was higher for children who had never received HAART versus those who had, at 89% and 86% versus 78% and 73% at 12 and 18 months of age, respectively.

## Discussion

We have investigated the therapeutic management, disease progression and survival rate in a birth cohort of vertically-infected children in Ukraine. Nearly half had started HAART by their most recent follow-up visit, with most of these children starting HAART recently (since 2007). In addition to HAART, 31% of children received prophylaxis and 4% received TB treatment. Substantial immunological improvement among children on HAART was evident and, among those with VL measurements available post-initiation, nearly three-quarters had achieved undetectable VL. Overall, almost a quarter of this cohort of infected children had developed AIDS, most in the first year of life, and around one in ten had died, at a median age of 6 months. Overall, survival increased significantly over the study period.

In Ukraine, ART became available for use in adults in 2001, with a somewhat slower roll-out of paediatric ART [15]. Treatment of HIV-infected children has been a priority of the national HIV programme, and the large-scale provision of ART for infected individuals started in 2004. Subsequently, the proportion of children with advanced HIV disease receiving ART increased, although it was estimated that at least a quarter of diagnosed children with advanced HIV disease nationally were not receiving treatment in 2007 [6]. Our findings indicate that more than half of the infected children had not received HAART and of those who received it, 86% had initiated treatment since 2007 onward. In addition, more than a third of moderately or severely immunosuppressed children had not yet started HAART, highlighting the remaining unmet need for treatment.

Overall median age at HAART initiation (18 months) was higher than reported in Western Europe [20], but has declined substantially, to around 9 months in recent years. This partly reflects the fact that in the early years of the study, diagnosis of infection in vertically-exposed infants depended on antibody testing at 15-18 months of age, with diagnosis through PCR testing only introduced in 2006. Another factor behind this trend has been the changes in paediatric treatment protocols over time: up to 2005, treatment of infected children was delayed until eligibility criteria were met. Current WHO guidance recommends treating all HIV-infected infants aged < 12 months with HAART, on the basis of results from the CHER trial and other studies on the clinical and immunological benefits of early initiation of HAART [7-9,21]. However, this approach requires prompt diagnosis of infected infants and rapid communication of the result to the family and caring physicians, which can be challenging in Ukraine where PCR testing is carried out in just three regional laboratories. Our findings highlight the importance of prompt early diagnosis in this population, as 15.5% of infants had rapid disease progression (developed AIDS or died) in their first year of life. Although PCR testing in Ukraine was available, only a quarter of infected children in our study born in 2007/08 had a PCR test before four months of age. Despite the remaining challenges with respect to early diagnosis of infected infants in Ukraine, the trend towards earlier diagnosis of HIV infection we have documented here, together with greater availability of HAART, are likely to explain the improved estimated survival rate among children in our cohort born in 2004/08 compared with those born earlier. Our finding of a substantially higher rate of AIDS-free survival among children who had never received HAART in comparison with those treated is most likely the result of confounding by indication, whereby children with immunological/clinical indications and thus with poorer survival prognosis were more likely to be prescribed HAART, but also more likely to have or to progress to AIDS or to die. Children on HAART here showed good virological response, and the 75% who achieved undetectable viral load was similar to that reported in other studies - for example 77.5% in Italy and 78% in the UK [20,22]. However, our results are limited by the fact that many children had only recently started HAART and thus had no post-initiation VL measurement available.

Cotrimoxazole is a widely available antibiotic, recommended by WHO and UNAIDS for prevention of opportunistic infections in individuals living with HIV/AIDS since April 2000[23]. Several guidelines recommend that cotrimoxazole should be started in all infants born to HIV-infected mothers at 6 weeks of age, continuing until confirmation of negative HIV status[24,25]. Results of one trial showed a 43% reduction in mortality and a 23% reduction in hospitalizations among HIV-infected children receiving cotrimoxazole compared to the control group[26]. Consequently, large-scale provision of cotrimoxazole to all infants born to HIV-infected mothers regardless of their age, CD4 count and resistance to the drug in low resource countries is recommended to prevent mortality and morbidity caused by PCP infection[26]. In our study PCP was the most common AIDS-defining disease followed by recurrent bacterial infection, consistent with other findings in Côte d'Ivoire, Thailand, the U.K., the U.S. and some European countries [11,22,27-29]. However, cotrimoxazole was provided to only 29% of children in our cohort. In addition, the majority (80%) of children who died with reported AIDS-defining diseases had infectious diseases including PCP but only 10% had received cotrimoxazole prophylaxis. Despite governmental policy for free cotrimoxazole coverage for HIV-infected children in Ukraine, our findings indicate that infected children do not have adequate access to prophylaxis; furthermore, anecdotal reports suggest that parents will be charged for the drug's purchase. The reason for low provision of cotrimoxazole to prenatally HIV-exposed infants in Ukraine remains unclear. However, anecdotal reports suggest contributing factors may include non-availability of cotrimoxazole in some AIDS Centers, a lack of paediatric formulations and problems with reimbursement to parents. In order to improve morbidity and mortality among vertically HIV infected children, there is an urgent need for scaling-up access to cotrimoxazole prophylaxis.

High prevalence of TB co-infection in HIV-infected people is a well recognised problem in resource-limited settings [30]. The incidence of TB in Ukraine was estimated at 82/100,000 in 2007 [31]. Although TB has been identified as the leading cause of death among HIV-infected population in Ukraine[6], only 15% of HIV-infected individuals with TB received TB treatment in 2006[6], reflecting old-fashioned approaches to TB control and weak linkages between HIV and TB services [32-34]. Children with HIV-infected parents have an increased likelihood of TB exposure [30], mainly due to the increased risk of acquiring TB from within their household; HIV-infected children may also be exposed to TB if staying on the same inpatient wards as HIV-infected adults with active TB [34]. Infants born to HIV-infected mothers do not receive BCG immunization in Ukraine; infants who serorevert are eligible for immunization but not infected children. Furthermore, TB-HIV coinfected children in Eastern Europe often have severe presentation of TB disease [32], In our study co-infection with extra-pulmonary M. tuberculosis infection was reported for 13% of children with AIDS; however, we may have underestimated the true prevalence of TB in the cohort. Optimizing TB prevention, screening and treatment strategies through integration of HIV-TB services are essential to reduce TB related morbidity and mortality among HIV-infected children.

We estimated that overall, 17% of children will have progressed to AIDS or death by age 12 months, increasing by only 4% over the next 6 months. This is consistent with early natural history studies, which demonstrated that 15-20% infants had an early severe disease (opportunistic infections, especially PCP, and encephalopathy), progressing rapidly to AIDS/death within their first year of life, while the remainder had more gradual disease progression, with around 3-5% progressing to AIDS or death annually [11,35]. Our finding of a significant improvement in AIDS-free survival over time reflects the roll-out of ART for infected children in Ukraine and the trend towards earlier diagnosis of infection in infants born to HIV-positive mothers.

Nearly a fifth of the infected children here were living in an institutional care setting. According to one national report, one-third of HIV-infected children with established status in Ukraine were under institutional care by the end of July 2007[6]. High rates of infant abandonment have also been reported from the Russian Federation [36,37], and in Western Europe earlier in the HIV epidemic, usually associated with maternal IDU and marginalised social status of many HIV-infected women [2,38,39]. Implementation of comprehensive PMTCT programmes may reduce risk of infant abandonment [2,4,40], and declining rates of abandonment have been documented concurrent with scale-up of PMTCT [40]. Since institutional care is associated with detrimental affect on all aspects of infant's development and HIV-infected infants are vulnerable to stigmatised care [37,40] strategies are needed for improving access to antenatal and other medical and social care for hard-to-reach pregnant women including IDUs. Furthermore, in our young (median age of 23 months) cohort of infected children, at least 4% had already experienced maternal death, highlighting a need for alternative social care for orphans and respite care for children with sick parents.

Unsurprisingly, PMTCT prophylaxis coverage among the mother-child pairs in this sub-cohort of infected children was lower than that seen for the whole cohort, which had 93% coverage in 2006 [4]; however, many of the children here were infected despite receipt of PMTCT prophylaxis, largely sdNVP or short-course ZDV, which are less effective than HAART. The current PMTCT policy in Ukraine recommends use of HAART as PMTCT prophylaxis as well as for maternal treatment, which is likely to result in a lower national MTCT rate, although this should be considered in the context of annually increasing numbers of HIV-infected women delivering in Ukraine[3]. MTCT rates remain elevated in some groups of women, particularly IDUs. One-third of mothers here reported an IDU history, higher than that seen in the ECS overall (around a fifth), reflecting the increased risk of intrapartum diagnosis and thus of MTCT among IDUs [4]. In order to achieve further and sustainable declines in MTCT and to optimise paediatric HIV treatment and care, it will be essential to implement non-stigmatised multidisciplinary services and continue to improve the coverage and quality of PMTCT interventions.

There are several limitations to this observational study, which has potential for both measured and unmeasured confounding. As an observational study in a real-life setting, there were some missing variables for some of the children, including CD4 and VL measurements. Information on adherence to therapy and viral resistance mutations was not collected, and thus we were unable to adjust for these potential confounders. The short median duration of HAART by the time of this analysis (6.6 months) is a further limitation; future analyses of data from this cohort will allow the longer-term impact of HAART to be addressed in addition to the impact of HAART started at younger ages and before progression to symptomatic disease. Follow-up of infected children from birth prevents under-estimation of mortality that can be a problem in non-birth cohorts and we expect our results to be broadly generalisable to the HIV-infected paediatric population in Ukraine.

## Conclusions

In conclusion, in this large birth cohort of vertically HIV-infected children in Ukraine, we have documented in a "real-life", operational setting that these children have a good response to HAART, which is being used earlier and more widely, reflecting the ongoing scale-up of early infant diagnosis and paediatric treatment nationally. The improvements in survival in more recent years of the study reflect these trends. Further research is needed to explore the issues of adherence, long term exposure to HAART and subsequent adverse effects in this population.

## Competing interests

The authors declare that they have no competing interests.

## Authors' contributions

CT, RM and SM contributed to study concept and CT, RM, IS and TP contributed to study design. RM, IS and TP were involved in the acquisition of data. SM and CT drafted the manuscript and SM performed the statistical analyses. The Ukraine European Collaborative Study Group contributed to the design and/or data collection for this study. All authors critically revised the manuscript for important intellectual content and read and approved the final manuscript.

## Funding

The ECS was a coordination action of the European Commission (PENTA/ECS 018865). Claire Thorne is supported by a Wellcome Trust Research Career Development Fellowship. Some of this work was undertaken at GOSH/UCL Institute of Child Health which received a proportion of funding from the UK Department of Health's NIHR Biomedical Research Centres funding scheme. The Centre for Paediatric Epidemiology and Biostatistics also benefits from funding support from the Medical Research Council in its capacity as the MRC Centre of Epidemiology for Child Health.

## Pre-publication history

The pre-publication history for this paper can be accessed here:

http://www.biomedcentral.com/1471-2431/10/85/prepub
